# Investigating the effectiveness of re-opening policies before vaccination during a pandemic: SD modelling research based on COVID-19 in Wuhan

**DOI:** 10.1186/s12889-021-11631-w

**Published:** 2021-09-07

**Authors:** Ying Qian, Wei Xie, Jidi Zhao, Ming Xue, Shiyong Liu, Lei Wang, Wanglai Li, Luojia Dai, Yuyang Cai

**Affiliations:** 1 Business School, University of Shanghai for Science & Technology, Shanghai, People’s Republic of China; 2 School of Public Administration, Faculty of Economics and Management, East China Normal University, Shanghai, People’s Republic of China; 3 School of Business Administration, Shanghai Lixin University of Accounting and Finance, Shanghai, People’s Republic of China; 4 Center for Governance Studies, Beijing Normal University, Zhuhai, 519087 People’s Republic of China; 5 Department of Information, Technology and Innovation, Antai College of Economics & Management, Shanghai Jiao Tong University, Shanghai, People’s Republic of China; 6 School of Public Health, Shanghai Jiao Tong University School of Medicine, Shanghai, People’s Republic of China

## Abstract

**Background:**

Lockdown policies were widely adopted during the coronavirus disease 2019 (COVID-19) pandemic to control the spread of the virus before vaccines became available. These policies had significant economic impacts and caused social disruptions. Early re-opening is preferable, but it introduces the risk of a resurgence of the epidemic. Although the World Health Organization has outlined criteria for re-opening, decisions on re-opening are mainly based on epidemiologic criteria. To date, the effectiveness of re-opening policies remains unclear.

**Methods:**

A system dynamics COVID-19 model, SEIHR(Q), was constructed by integrating infection prevention and control measures implemented in Wuhan into the classic SEIR epidemiological model and was validated with real-world data. The input data were obtained from official websites and the published literature.

**Results:**

The simulation results showed that track-and-trace measures had significant effects on the level of risk associated with re-opening. In the case of Wuhan, where comprehensive contact tracing was implemented, there would have been almost no risk associated with re-opening. With partial contact tracing, re-opening would have led to a minor second wave of the epidemic. However, if only limited contact tracing had been implemented, a more severe second outbreak of the epidemic would have occurred, overwhelming the available medical resources. If the ability to implement a track-trace-quarantine policy is fixed, the epidemiological criteria need to be further taken into account. The model simulation revealed different levels of risk associated with re-opening under different levels of track-and-trace ability and various epidemiological criteria. A matrix was developed to evaluate the effectiveness of the re-opening policies.

**Conclusions:**

The SEIHR(Q) model designed in this study can quantify the impact of various re-opening policies on the spread of COVID-19. Integrating epidemiologic criteria, the contact tracing policy, and medical resources, the model simulation predicts whether the re-opening policy is likely to lead to a further outbreak of the epidemic and provides evidence-based support for decisions regarding safe re-opening during an ongoing epidemic.

**Keyords:**

COVID-19; Risk of re-opening; Effectiveness of re-opening policies; IPC measures; SD modelling.

## Background

Several ways exist to fight an epidemic caused by a novel virus. Vaccination is admittedly the most effective approach because it can make the human body immune to the virus and thus break the chain of transmission. However, the development and implementation of a new vaccine requires a relatively long time. In the case of coronavirus disease 2019 (COVID-19), even with accelerated timelines for development, the world’s first COVID-19 vaccine did not appear until August 2020, 9 months after the report of the first COVID-19 case. Currently, with dozens of COVID-19 vaccines in clinical trials and many nations starting to vaccinate their populations, there is still no certainty about when COVID-19 vaccines will become widely available around the world [1].

In the absence of a vaccine, non-pharmaceutical infection prevention and control (IPC) measures can be reliable “weapons” in the fight against the virus. These include stay-at-home or shelter-in-place lockdowns. A lockdown usually includes the closure of schools and businesses, movement restrictions, international travel restrictions, and geographic area quarantines [2]. The highly contagious nature of COVID-19 forced a number of jurisdictions around the world to apply the strictest form of movement restriction, complete lockdown. The effectiveness of timely lockdowns has been indicated by many quantitative modelling studies [3–5]. For example, Lai et al. [4] found that one-, two- or three-week delays in implementing a lockdown in Wuhan, China, would have led to a 3-fold, 7-fold or 18-fold increase in the number of cases, respectively.

However, the shutdown of an administrative district has enormous social and economic costs, including ripple effects such as the loss of income, unemployment, anxiety and suicide [6]. Governments that applied lockdown policies planned to resume social and economic activities as soon as the pandemic was under control. However, re-opening comes with the risk of a resurgence in infections because it leads to increases in population mobility and contact rates. Contact between individuals at bars, restaurants, and shops has been found to drive the number of infections [7]. Recent modelling studies have suggested that relaxing restrictions before it is safe to do so could have disastrous consequences [8]. It remains unclear when is the proper time to re-open without the protection of an effective, widely available vaccine.

**Fig. 1:**
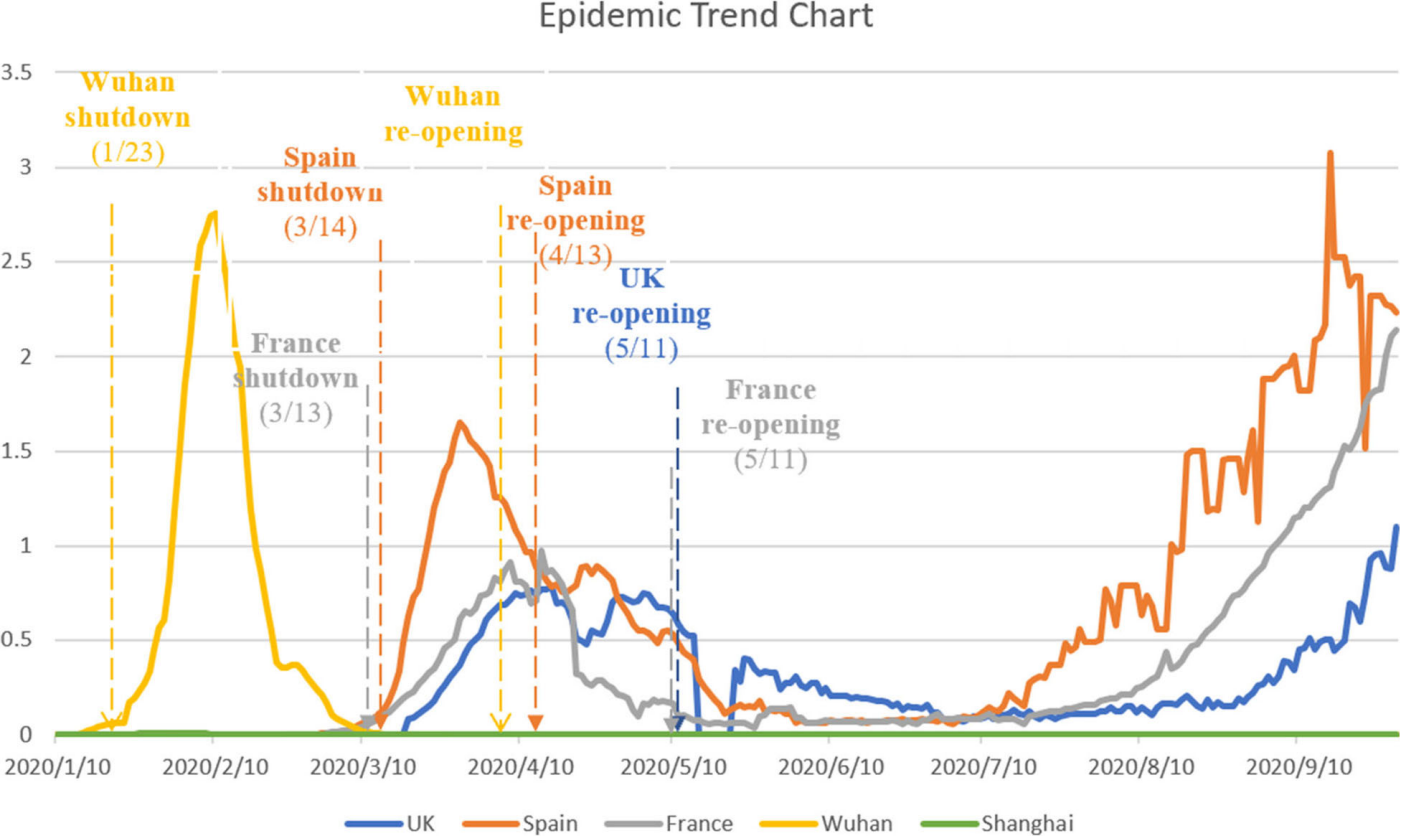
Number of new COVID-19 cases per 10,000 people by jurisdiction

Different governments have applied lockdowns of different lengths and intensities. Figure 1 summarizes the dates on which the governments of the UK, France, Spain, Shanghai, and Wuhan announced lockdowns and re-openings, as well as the number of daily new COVID-19 cases per 10,000 people over time. The consequences of re-opening have varied across countries. Some experienced a severe second outbreak of the epidemic, in which the number of daily new cases resurged and reached a peak value higher than that of the previous wave. Others had a relatively safe resumption of social and economic activities, in which only sporadic or small clusters of cases occurred after re-opening [9]. It can also be observed in Fig. 1 that a longer lockdown, i.e., a later re-opening, did not guarantee a reduced severity of the second outbreak of COVID-19. Therefore, policies aimed at facilitating a safe re-opening need further investigation.

The World Health Organization (WHO) has outlined six criteria that each country should meet before lifting restrictions: 1) transmission is controlled, 2) test-trace-isolation capacities are in place, 3) outbreak risks are minimized in high-vulnerability settings such as nursing homes, 4) preventative measures are in place in workplaces and schools, 5) the risks of exporting and importing cases are managed, and 6) communities are educated and empowered to make the necessary adjustments [2]. However, government decision-makers often emphasize the first category, relying only on epidemiologic criteria, i.e., the daily number of new COVID-19 cases. For example, Wuhan followed a strict epidemiologic criterion – the number of new confirmed cases must have decreased continuously over the previous 14 days and reached zero. Conversely, national and state governments in U.S. adopted various criteria, including no more than 1 case per 10,000 people in the last 14 days and no more than 25 cases per 100,000 people in the last 14 days.

Insufficient consideration has been given to the effects of other measures, especially contact tracing policies and their real-world implementation. In an assessment of countries attempting to follow the WHO recommendations for rolling back their lockdowns, the Oxford research group found that the largest variation among countries has centred on the testing and tracing metrics [10]. This is partly because the impact of track-and-trace measures on re-opening is difficult to quantify.

To address this gap, we developed an epidemiological model based on a case study of COVID-19 in Wuhan and investigated the effectiveness of the re-opening policies considering the epidemiologic criteria and track-trace-quarantine measures. This study will produce a testbed for IPC measures and present evidence-based experiences with re-opening to assist with decision-making in controlling the spread of a new virus when a vaccine is not available.

## Methods

### Data

We use the COVID-19 outbreak in the city of Wuhan, China, as the case for this study. Wuhan was shut down on Jan. 23rd, 2020, and has experienced a remarkably safe resumption of social and economic activities since Mar. 16th, 2020. The typicality and representativeness of the COVID-19 outbreak in Wuhan as the research object of a case study have been illustrated by numerous publications [3, 11, 12], but to the best of our knowledge, few studies have addressed the risk associated with re-opening during the COVID-19 pandemic. Epidemiological data for COVID-19 in Wuhan come from publicly released data collected by the National Health Commission of the People’s Republic of China and the Health Commission of Hubei Province (in which Wuhan is located) [13, 14]. The dataset includes (1) daily counts of newly confirmed cases, fatalities, and recoveries; (2) cumulative counts of confirmed cases, deaths, and recoveries; (3) the number of identified close contacts; and (4) the number of patients receiving medical treatment in hospitals. The data cover the time period from Jan. 10th (when the first case was confirmed) to Apr. 30th (when the number of newly confirmed cases stabilized at 0). From these data, it is possible to estimate the rates of change needed to parameterize a dynamic model. This research did not access individual patient data; thus, ethics approval and patient informed consent were not required.

### SD modelling Methods

We developed a model of COVID-19 transmission in Wuhan using system dynamics (SD). The essence of SD modelling is to understand how the behaviour of complex systems changes over time by recognizing the system structure with causal feedback loops [15] and then codifying them into stock and flow diagrams [16]. The simulation of SD models illustrates how multiple feedback loops interact over time and lead to time-based behaviour. Changes in the behaviour patterns of complex systems are often accompanied by changes in loop dominance, providing new insights into complex behaviour [17]. Through model simulation, it is possible to investigate how a system’s behaviour evolves and identify leverage points for policies. It is also useful when real-world complexity surpasses the ability to create closed-form solutions. Thus, SD models can be used as a micro-environment to test the effectiveness of various policies [18].

### Model structure

The SEIR framework, which models the flows of people between four states, namely, susceptible (S), exposed (E), infectious (I), and recovered (R), has been widely used to study the transmission of disease [19, 20]. However, the original model does not explicitly consider the impact of IPC measures taken by the government, such as quarantining the contacts of people with newly confirmed cases and isolating patients with confirmed cases in hospitals for medical treatment. In addition, asymptomatic individuals infected with SARS-CoV-2 (the virus that causes COVID-19) are infectious [21]. This is contrary to the original assumption that individuals in the “exposed” (latent) state, i.e., those individuals who have been infected but are currently asymptomatic, are not infectious. Therefore, we extended the original SEIR framework to model three new elements: 1) the infectiousness of asymptomatic exposed individuals, 2) hospitalized patients, and 3) the quarantine of close contacts of known infected persons.

Thus, the total population in Wuhan (N) was further stratified to include the quarantined susceptible (Sq), quarantined exposed (Eq), quarantined infected with symptoms (Iq), and hospitalized (H) subgroups in addition to the traditional S, E, I, and R subgroups.

$$ N=S+{S}_q+E+{E}_q+I+{I}_q+H+R+{R}_I+D+{D}_I $$

**Fig. 2:**
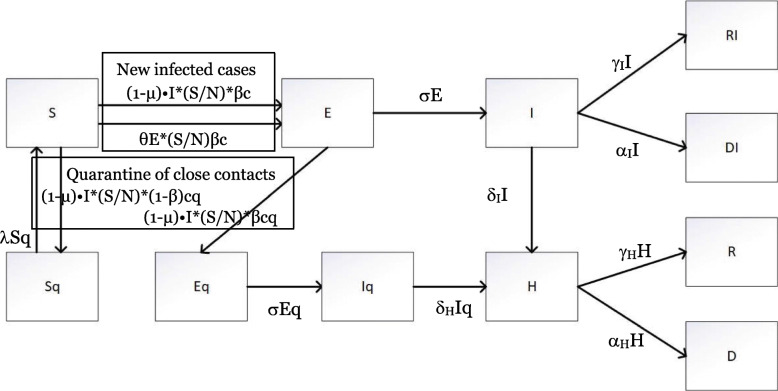
Structure of the SEIHR(Q) model

Figure 2 shows the structure of our model, called Susceptible-Exposed-Infected-Hospitalized-Removed with and without Quarantine (SEIHR(Q)).

Sq and S represent the susceptible population with and without quarantine, respectively.

Eq and E represent the infected population during the incubation period with and without quarantine. One feature of COVID-19 is that the latent population can also spread the virus, which turns S into E.

Iq and I represent the infected population that develops symptoms with and without quarantine.

H represents the population in hospitals. In general, infected people will be admitted to the hospital after testing. Special cases include infected individuals (especially those with minor symptoms) who might not be willing to stay in the hospital or cannot be received by the hospital, especially at times when medical resources are inadequate. In the case of Wuhan, at the beginning of the outbreak, when testing capacity was inadequate, the infected population had to wait several days before receiving their test results and being admitted to a hospital.

D and DI represent the population that died in the hospital or outside a hospital.

R and RI represent the population that recovered from hospital treatment or recovered without hospital treatment.

The upper part of the model, where S is converted to E, then to I, and then to recovery or death, is similar to the traditional SEIR model. Two extensions are implemented: 1) in COVID-19, E can also transmit the disease to S in a similar way as infected group I but with a lower proportion θ, and 2) the Wuhan government set up specialized fever clinics to care for people with a fever in an effort to isolate those with suspected COVID-19 cases during the diagnosis and treatment process and remove them from the possible transmission chain as early as possible. Assuming that μ of the infected with symptoms population I are isolated at the fever clinic before their infection is confirmed, the number of effective sources of infection would be (1-μ) •I. Therefore, individuals with new infections, i.e., individuals in group S transmitting to group E is given by (1 − *μ*) ∗ *I* ∗ (*S*/*N*) ∗ *βc* + *θE* ∗ (*S*/*N*)*βc*, where the transmission probability is β and the contact rate is c.

Movements from S to Sq and E to Eq represent the impact of the track-and-trace measures on close contacts of individuals with cases of COVID-19. Close contacts refer to people who have been in close contact with an infectious COVID-19 patient and have not been properly protected [22]. In the case of Wuhan, whenever a new case is detected, staff of the Centre for Disease Control and Prevention (CDC) conduct a detailed and rigorous epidemiological investigation to learn about the patient’s activities and suspicious contact during the 14 days before the onset of the disease. Based on the recommendations of the Chinese clinical guidelines for COVID-19 from the National Health Commission of the People’s Republic of China [23], close contacts undergo a compulsory 14-day quarantine in single rooms at their own expense. They either develop confirmed cases during the quarantine period (move from Eq to Iq) or leave quarantine after the period (move from Sq to S).

Supposing that the proportion of close contacts of a confirmed case that can be identified and quarantined is q, then the rates of E moving to Eq and S moving to Sq are given by (1 − *μ*) ∗ *I* ∗ (*S*/*N*) ∗ *βcq* and (1 − *μ*) ∗ *I* ∗ (*S*/*N*) ∗ (1 ‐ *β*)*cq*, respectively.

Therefore, the number of susceptible individuals in group S is.

$$ S(t)=S(0)+{\int}_0^t\left(\lambda {S}_q-\left(1-\mu \right)I\left(\tau \right)\beta cS\left(\tau \right)/N\left(\tau \right)-\theta E\left(\tau \right)\beta cS\left(\tau \right)/N\left(\tau \right)-\left(1-\mu \right)I\left(\tau \right)\left(1-\beta \right) cqS\left(\tau \right)/N\left(\tau \right)\right){d}_{\tau } $$

The number of susceptible cases under quarantine is given by

$$ {S}_q(t)={S}_q(0)+{\int}_0^t\left(\left(1-\mu \right)I\left(\tau \right)\left(1-\beta \right) cqS\left(\tau \right)/N\left(\tau \right)-\lambda {S}_q\left(\tau \right)\right){d}_{\tau } $$

People move from E to I at the rate of σ. The number of exposed individuals in group E is

$$ E(t)=E(0)+{\int}_0^t\left(\left(1-\mu \right)I\left(\tau \right)\beta c\left(1-q\right)S\left(\tau \right)/N\left(\tau \right)+\theta E\left(\tau \right)\beta c S\left(\tau \right)/N\left(\tau \right)-\sigma E\left(\tau \right)\right){d}_{\tau } $$

The number of exposed individuals under quarantine is given by

$$ {E}_q(t)={E}_q(0)+{\int}_0^t\left(\left(1-\mu \right)I\left(\tau \right)\beta cqS\left(\tau \right)/N\left(\tau \right)-\sigma {E}_q\left(\tau \right)\right){d}_{\tau } $$

Individuals with severe symptoms go to hospitals where they are isolated and receive medical treatment at a rate of δI. Infected individuals without symptoms or with mild symptoms do not need to go to the hospital and move to the recovery group RI or death group DI at rates of γ_I_ and α_I,_ respectively. Thus, the number of these individuals in I is given by

$$ I(t)=I(0)+{\int}_0^t\left(\sigma E\left(\tau \right)-{\delta}_II\left(\tau \right)-{\alpha}_II\left(\tau \right)-{\gamma}_II\left(\tau \right)\right){d}_{\tau } $$

and the number of cases in group RI and DI are given by

$$ RI(t)= RI(0)+{\int}_0^t\left({\gamma}_II\left(\tau \right)\right){d}_{\tau } $$

$$ DI(t)= DI(0)+{\int}_0^t\left({\alpha}_II\left(\tau \right)\right){d}_{\tau } $$

People move from Eq to group Iq at a rate of σ and receive medical treatment in the hospital at a rate of δ_H_. Therefore, the number of quarantined infected individuals is given by

$$ {I}_q(t)={I}_q(0)+{\int}_0^t\left(\sigma {E}_q\left(\tau \right)-{\delta}_H{I}_q\left(\tau \right)\right){d}_{\tau } $$

Then, infected individuals in the hospital move to the recovery group R or the death group D at rates of γH and αH, respectively. Accordingly, the number of infected individuals in hospitals is

$$ H(t)=H(0)+{\int}_0^t\left({\delta}_H{I}_q\left(\tau \right)+{\delta}_II\left(\tau \right)-{\alpha}_HH\left(\tau \right)-{\gamma}_HH\left(\tau \right)\right){d}_{\tau } $$

The number of individuals who were hospitalized and then recovered or died are

$$ R(t)=R(0)+{\int}_0^t\left({\gamma}_HH\left(\tau \right)\right){d}_{\tau } $$

$$ D(t)=D(0)+{\int}_0^t\left({\alpha}_HH\left(\tau \right)\right){d}_{\tau } $$

The parameters, their definitions, their values and the validity of their values are listed in Table 1. To estimate the dynamics of an epidemic, a couple of similar pairs of concepts with different definitions, including the incubation period [30] and the latent period [33], the serial interval and the generation time [34], have been proposed as key parameters. Besides the slight differences in concepts, a number of scientific papers reported various values for these parameters. Griffin et al. conducted a literature review of these works and summarised different estimates of these parameters for COVID-19 [34]. Following previous studies including papers in top journals, we choose to use the incubation period, which is the time between infection and the onset of symptoms [30] with a mean/median value of 5.2 days [11, 27–30].

**Table 1:** Parameter settings in the SEIHR(Q) model

Parameter and definition	Value	Source and Explanation
c: Contact rate	14.5	Prior to the implementation of the Level-1 emergency response on January 23 [13, 24], the contact rate was approximately 14.5 [25]. After that, the contact rate dropped dramatically and stabilized at a low rate of 4. Since the resumption of work, the value has gone up gradually.
β: Probability of transmission	0.035	The reproduction number R can be calculated as the transmission rate multiplied by the transmission time, which in this model is βc/σ. In Wuhan, the initial R0 was estimated to be approximately 2.67 [26, 27]. Therefore, β can be calculated to be 0.035
σ: Transition rate	0.19	The mean incubation period is 5.2 days [11, 27–30].
q: Quarantined rate of exposed individuals	0	Initialized as 0 and gradually increased to 0.7 as Level-1 emergency response was implemented [11, 13].
λ: Rate of return from Sq to S	1/14	Estimated based on the recommendation of the Shanghai Municipal Health Commission [31].
δ_I_: Transition rate of individuals in I to the hospitalized group	0.12	The initial value taken from the reference [11] quickly dropped due to abundant medical resources in Wuhan and the acceleration of nucleic acid testing.
δ_H_: Transition rate of individuals in Iq to the hospitalized group	1	Quarantined infected individuals were admitted to the hospital immediately after nucleic acid testing.
μ: Isolation rate of patients at fever clinics	0	No isolation at a fever clinic in Wuhan at the beginning. With the policy of stricter isolation at fever clinics, the value increased to 0.4 and then 0.7 [32].
γ_I_: Recovery rate of individuals in I	0.07	Individuals with mild symptoms or an asymptomatic infection recovered within 14 days on average [11, 12].
γ_H_: Recovery rate of individuals in H	0.01	Model calibration
α_I,_α_H_: Death rate	0.004	Model calibration
θ: Infectious weight in incubation period	0.5	Consistent with the reference [11, 12].
q: Percentage of close contacts who are quarantined	0	Initialized as 0 and gradually increased to 0.7 as Level-1 emergency response was implemented [13, 32]..

The assumptions of this model include:

(1) Population movements between Wuhan and other parts in China as well as foreign countries were not allowed due to the lockdown policy; therefore, N was fixed. Population changes from births and from deaths due to non-COVID-19 causes were not included in the model because of the short time horizon of this study (less than a year).

(2) Patients who recovered from COVID-19 were assumed to be non-infectious and not at risk of a second infection [35].

(3) Individuals with suspected COVID-19 cases were quarantined or isolated in single rooms and did not come into contact with non-quarantined/isolated individuals. This assumption was made based on the recommendations of the Chinese clinical guidelines for COVID-19 [23]. In Wuhan, few confirmed cases resulted from contact with quarantined individuals. Therefore, the model assumed that quarantined or isolated individuals did not infect any other individuals.

(4) Work resumption occurred in stages, and people’s contact rate gradually increased after re-opening. In addition, even when full re-opening had been implemented, people usually reduced unnecessary outdoor activities during the ongoing pandemic. Thus, the contact rate after re-opening would not be as high as that before the COVID-19 pandemic. As supported by the Baidu migration data [36], 90% of the normal contact rate before the COVID-19 pandemic was adopted in the model.

### Model validation

We constructed the SEIHR(Q) model using Vensim 8.09, developed by Ventana Systems in the United States. Vensim provides a flexible way of building system dynamics simulation models and offers tools for improving the quality of the models, such as unit check and sensitivity tests. The model calibration function in Vensim helps estimate parameters without precise numerical values.

**Fig. 3:**
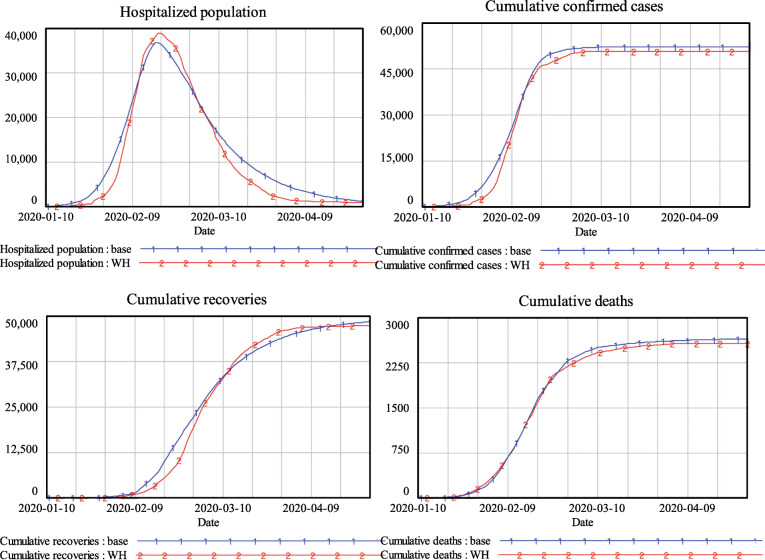
Simulation results and historical data from the government

The validity of the model structure lies in the fact that the SEIR model has been widely used for the simulation of the spread of epidemics. Moreover, the simulation results of SEIHR(Q) are highly consistent with historical data released by the Heath Commission of Hubei Province, as shown in Fig. 3. Variables such as the number of existing cases in the hospital, the number of cumulative confirmed cases, the number of cumulative deaths, and the number of cumulative recoveries reproduce historical values. The simulation results for validation stopped in early June, as there were no new cases in Wuhan after Mar. 16th, the hospitalized population approached zero at the end of April, and the cumulative number of confirmed cases stabilized thereafter.

Table 2 compares the historical and simulated peak values of the hospitalized population as well as the peak dates. The simulated peak date of hospitalized cases is 2 days ahead of the historical data, and its peak value shows a − 7.13% difference from the historical data. For cumulative confirmed cases, cumulative recoveries and cumulative deaths on Apr. 30th, the differences were only 5.09, 3.73 and 0.12%, respectively. Therefore, the validity of the SEIHR(Q) model for COVID-19 Wuhan is confirmed, and the model has face validity for running policy simulations.

**Table 2:** Comparing simulated results with historical data

	Simulated	Historical	Difference (%)
Hospitalized peak value	36,820	38,970	−5.52%
Hospitalized peak date	Feb 17th	Feb 18th	−1 days
Cumulative confirmed cases on Apr. 30th	52,300	50,860	2.83%
Cumulative recovery on Apr. 30th	48,560	47,390	2.47%
Cumulative deaths on Apr. 30th	2650	2579	2.75%

## Results: policy simulation

In this study, we focused our investigation on two IPC measures: epidemiologic criteria for re-opening and intervention measures targeting close contact tracking, tracing and quarantining. The epidemiological criteria for re-opening are mostly concerned with the number of new confirmed cases expressed as a proportion of the population. For example, in the US in general and specifically in the state of California, two criteria have been used: one is fewer than 25 new confirmed cases in a population of 100,000, and the other is fewer than 1 new confirmed case in a population of 10,000. In Wuhan, the criterion was that the number of new confirmed cases had reached zero and there had been a continuous decrease in case numbers over the previous 14 or more days. As COVID-19 has an incubation period up to 14 days, the most conservative re-opening criterion would be to wait another 14 days after the number of new confirmed cases reaches zero.

Tracking and tracing close contacts of individuals with confirmed cases is very labour intensive to implement. Human resources, technological capacity, and the degree of cooperation from citizens will impact the percentage of detected close contacts. The most important factor determining the success of this measure is the degree to which governments are willing to emphasize and put effort into contact tracing.

### Exploring the contact tracing policy

The Wuhan government put a tremendous effort into contact tracing to prevent and control the spread of COVID-19 as work was resumed. The question to be investigated is how the epidemic would have evolved if Wuhan had changed its contact tracing policy, resulting in different percentages of close contacts, q, being identified and quarantined. The parameter settings are shown in Table 3.

**Table 3:** Different intensities of the contact tracing policy

Close contact tracing policy	Scenario name	q (Percentage of contacts traced)
Limited contact tracing	S1	20%
	S2	30%
Partial contact tracing	S3	40%
	S4	50%
Comprehensive contact tracing	S5	60%
	S6	80%

**Fig. 4:**
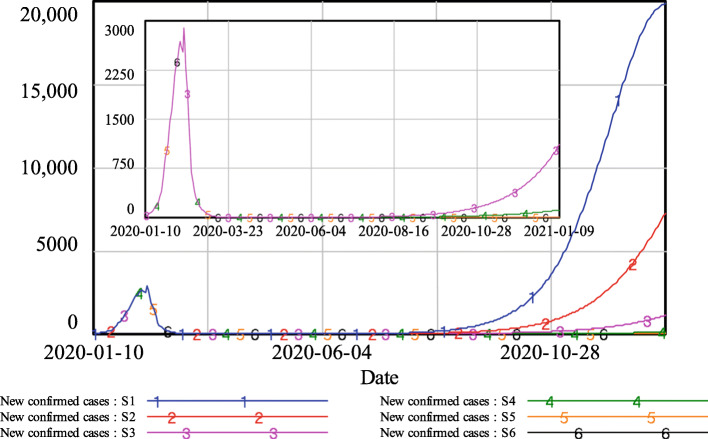
Simulation results of new confirmed cases under different levels of track-and-trace

The model simulations in Fig. 4 show that with comprehensive contact tracing, i.e., with q reaching 60% or above, the evolution of the epidemic after re-opening was similar, converging to zero new confirmed cases. Partial contact tracing led to an increase in the number of new confirmed cases. When q was 50%, the new confirmed cases slowly increased to approximately 113 at the end of the simulation. However, when q was reduced to 40%, the new confirmed cases increased to triple digits in mid-October and reached approximately 1086 new confirmed cases every day by the end of the simulation. Limited contact tracing caused a sharp increase in newly confirmed cases. When q was 30%, the number of new confirmed cases increased to more than 100 at the end of August and finally reached more than 7000 new confirmed cases every day. In the case when q was only 20%, the situation became even more severe: the new confirmed cases reached triple digits in early August and reached approximately 20,000 at the end of the simulation.

**Fig. 5:**
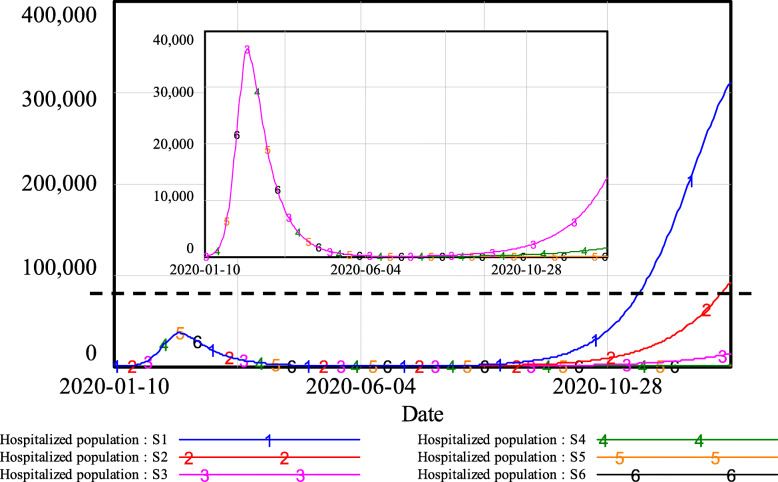
Simulation results of the hospitalized population under different levels of track-and-trace

As shown in Fig. 5, under comprehensive contact tracing, the hospitalized population remained very low, with fewer than 200 people. For partial contact tracing, when q was 50%, the hospitalized population increased slightly, reaching approximately 1500 at the end of simulation; when q was 40%, the hospitalized population exceeded four digits in early October and reached almost 14,000 people in the end. Under limited contact tracing, a severe second outbreak of the epidemic occurred. When q was 30%, the hospitalized population increased to more than 1000 in late August and finally reached approximately 91,700. With q at 20%, the hospitalized population quickly increased to four digits in early August and finally climbed to more than 310,000 people.

The total number of beds in medical and health institutions is regarded as a key indicator of medical care capacity, as it limits the number of infected patients the local hospital institutions can hold (1). According to publicly released reports, the total number of beds in Wuhan medical and health institutions in 2019 was 99,400 [37], as marked by the dotted line in Fig. 5. Simulation results show that when q was larger than 40%, existing medical care resources would be sufficient for receiving all COVID-19 cases and providing medical treatment. However, with a trace and quarantine proportion less than 30%, the local medical care capability would be insufficient, which would lead to a disaster in which patients would not be able to receive proper treatment and could not be admitted to the hospital, promoting the further spread the virus to the susceptible population.

**Fig. 6:**
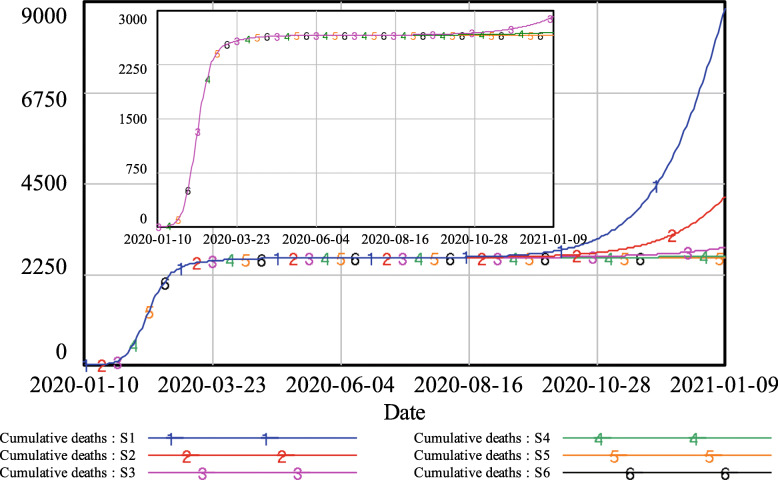
Simulation results of cumulative deaths under different levels of track-and-trace

Figure 6 shows the results for cumulative deaths. With comprehensive contact tracing, the number of deaths stabilized at approximately 2600 persons. With partial contact tracing, the number of deaths increased slightly, reaching approximately 2700 and 2900 when q was 50 and 40%, respectively. Under limited contact tracing, the number of deaths showed a sharp increase. When q equalled 30%, the number of deaths exceeded 4100 at the end of the simulation; the consequence of q being only 20% was even more severe, with the number of deaths climbing to more than 8800 people.

### Addressing safe epidemiological criteria with various intensities of contact tracing policy

Wuhan resumed work on Mar. 16th, after there had been more than 14 days characterized by a successive decrease in new confirmed cases and at which point the number of new confirmed cases had reached zero. The proper criteria for re-opening under different levels of implementation of the contact tracing policy remain unclear. The question of interest is what the consequences would have been if Wuhan had re-opened earlier or later. For a given jurisdiction, the ability to implement a track-trace-quarantine policy may not be adjustable within a short time period. Therefore, a more general research question is, with a given q, how would the epidemic evolve if the authorities decided to re-open earlier or later. Here, we simulated the development of the epidemic under different re-opening criteria at four different levels of q. The parameter settings are shown in Table 4.

**Table 4:** Epidemiological criteria for re-opening

Criteria	Definition	Re-opening Date in Wuhan’s case
C1	The number of new confirmed cases was fewer than 25 per 100,000 population over the past 14 days	Feb. 27th
C2	The number of new confirmed cases was fewer than 1 per 10,000 population over the past 14 days	Mar. 3rd
C3	Successive decrease in new confirmed cases over the past 14 days, reaching zero	Mar. 16th
C4	Zero new confirmed cases over the past 14 days	Mar. 30th

1. Re-opening under the condition that 60% of close contacts can be traced and quarantined

**Fig. 7:**
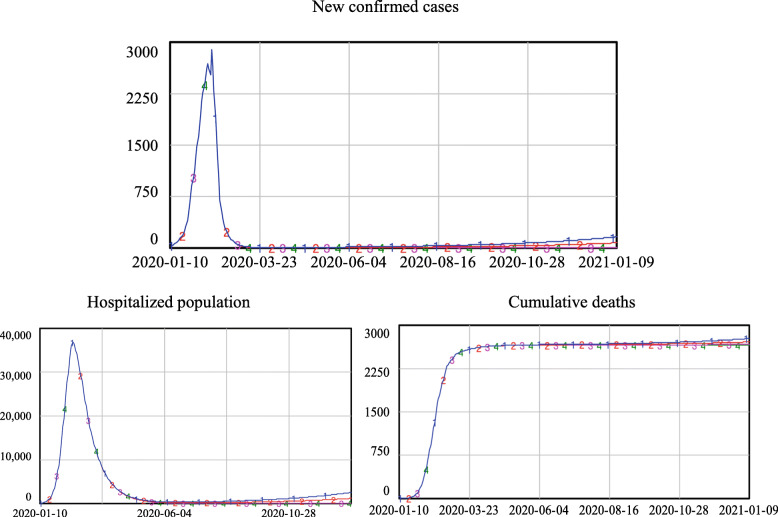
Simulation results if the quarantine level equals 60%

As shown in Fig. 7, if 60% of the close contacts could be traced and quarantined, no second wave of the epidemic would appear after re-opening, regardless of which epidemiological criteria were applied. Under the most relaxed criteria, C1, which would have led to the re-opening starting on Feb. 27th, the number of newly confirmed cases would only be 157 people at the end of the simulation. This demonstrates that, provided that comprehensive contract tracing was in place in Wuhan, full re-opening could have taken place as early as Feb. 27th.

2) Re-opening under the condition that 50% of close contacts can be traced and quarantined

**Fig. 8:**
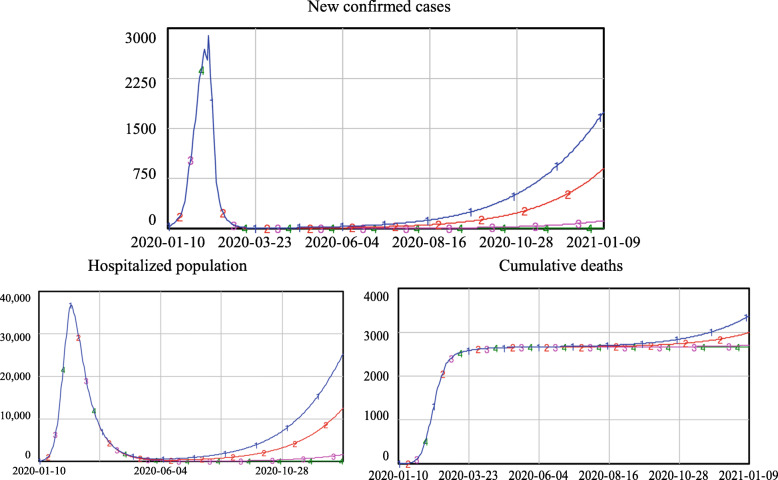
Simulation results if the quarantine level equals 50%

As shown in Fig. 8, under the condition that 50% of the close contacts could be traced and quarantined, both C3 and C4 would lead to a safe re-opening; the number of new confirmed cases and the number of hospitalized cases did not increase after the re-opening. However, criteria C1 and C2 would result in increases in new confirmed cases, although not as much as during the first outbreak. The number of new confirmed cases reached 1700 and 900 for criteria C1 and C2, respectively, at the end of the simulation, and the hospitalized population reached more than 22,000 and 11,000, respectively. Deaths stabilized at approximately 2600 with criteria C3 and C4, but with criteria C1 and C2, the number of deaths climbed to approximately 3200 and 2900, which represent increases of 18 and 12%, respectively.

3) Re-opening under the condition that 40% of close contacts can be traced and quarantined

**Fig. 9:**
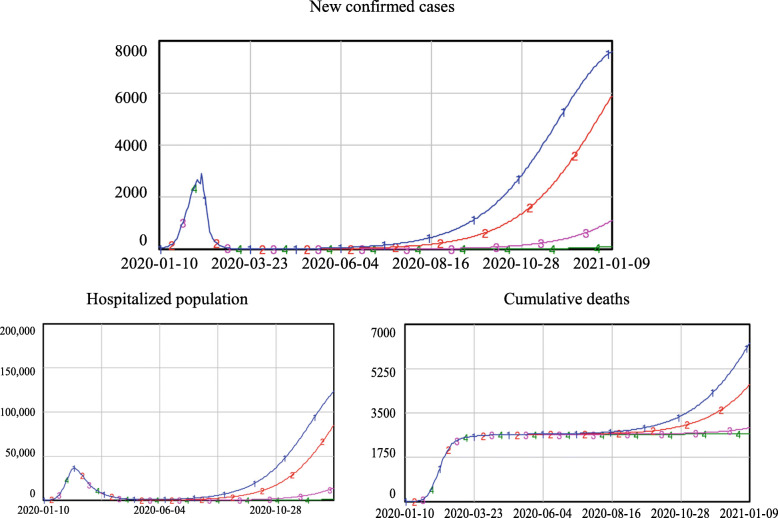
Simulation results if the quarantine level equals 40%

Figure 9 shows that if 40% of the close contacts could be traced and quarantined, the use of C1 and C2 would lead to a second outbreak of the epidemic; new confirmed cases would sharply increase until they exceeded 7000 and 5000, respectively. The hospitalized population would exceed 123,000 and 85,000 using criteria C1 and C2, respectively. Since the total number of beds in Wuhan medical and health institutions is 99,400 [37], the use of C1 would exhaust the available medical resources. Compared to the number of deaths in the first wave (approximately 2600), the number of deaths would be more than doubled using criteria C1 (approximately 6200) and increased by more than a 50% using C2 (approximately 4600).

4) Re-opening under the condition that 30% of close contacts can be traced and quarantined

**Fig. 10:**
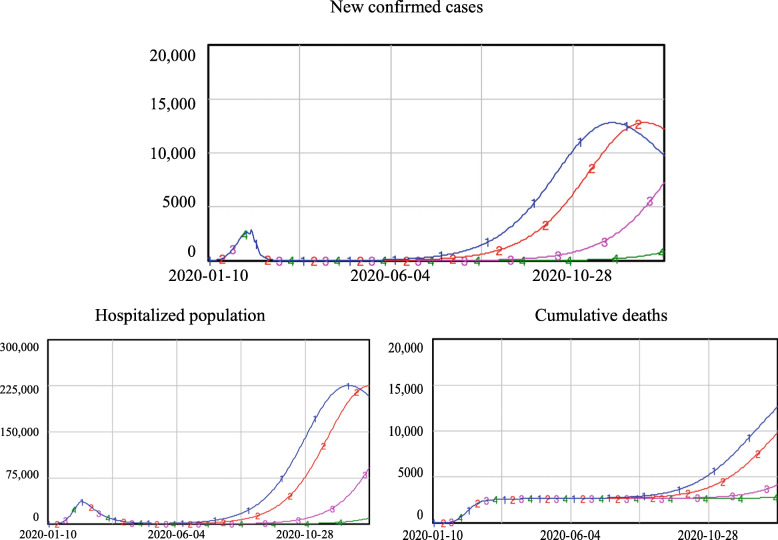
Simulation results if the quarantine level equals 30%

Figure 10 shows that if 30% of close contacts could be traced and quarantined, C1 and C2 would lead to a severe second outbreak of the epidemic; new confirmed cases would sharply increase and peak at approximately 13,000 per day. Furthermore, the hospitalized population would peak at approximately 220,000, a number well beyond the capacity of existing medical resources, which in the real world would lead to a collapse of the medical system without the influx of new resources. Using C3, the peak number of new confirmed cases and the hospitalized population in the second wave would be slightly higher than those in the first wave. Using C4, there would be limited increases in the new confirmed cases and hospitalized population.

5) Re-opening under the condition that 20% of close contacts can be traced and quarantined

**Fig. 11:**
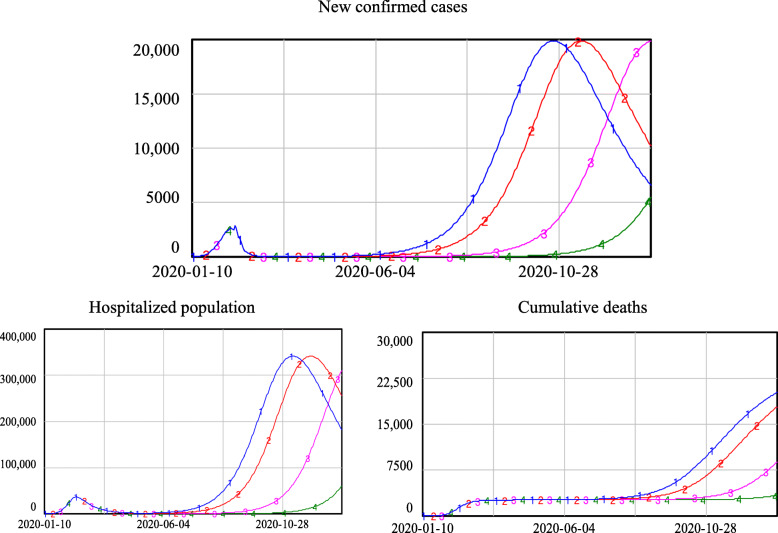
Simulation results if the quarantine level equals 20%

Figure 11 shows that if 20% of the close contacts could be traced and quarantined, C1, C2 and C3 would lead to a severe second outbreak of the epidemic; new confirmed cases would sharply increase and peak at approximately 20,000 per day. The peak value of the hospitalized population would climb to over 340,000 and far exceed the capacity of available medical resources. Even C4 would lead to a second severe outbreak, causing the number of new confirmed cases, hospitalized population and cumulative deaths to exceed their first wave peaks.

## Discussion

From the model simulation results, it is obvious that some re-opening policies would lead to a severe second outbreak, while others would not. Based on the severity of the second outbreak, we classified the risk of re-opening into the following categories: (1) no-risk/safe, meaning that there was almost no observed increase in the number of new cases after re-opening; (2) low risk, which corresponds to a slight increase in new confirmed cases, but a lower peak in the second wave than in the first; (3) high risk, which corresponds to a sharp increase in new infected cases, generating a higher peak in the second wave than in the first; and (4) unacceptable risk, in which case not only would the second wave have a higher peak value but the hospitalized population would exceed the hospital capacity, which implies a collapse of the medical system if new resources were not supplied.

In the case of Wuhan, the following conclusions can be drawn from the simulation results shown in Figs. 4, 5, and 6. With comprehensive contact tracing, there would have been almost no risk of re-opening and returning to normal daily activities. With partial contact tracing in place, re-opening in Wuhan would have led to a low-risk situation. However, if only limited contact tracing had been implemented, the risk resulting from re-opening would have been unacceptable.

To examine situations in which the ability to implement the track-trace-quarantine policy is fixed within a short time period, this study conducted additional simulations to reveal the risk of re-opening under various epidemiological criteria with a given proportion of close contacts being traced and quarantined. We developed a matrix to evaluate the risk of re-opening under different re-opening policies, as shown in Table 5. More specifically, when q was 60% or above, it would be safe to re-open, even with the most relaxed epidemiological criterion. When q was 50%, the relaxed epidemiologic criteria (C1, C2) would lead to a minor level of risk, generating a second wave of the epidemic that would not be not as severe as the first wave, while the strict criteria (C3, C4) would lead to no risk after re-opening. Under the condition that 40% of close contacts were traced and quarantined, the risk of re-opening varied across epidemiological criteria. The most relaxed epidemiological criterion (C1) would cause disaster, as the soaring number of infections would exceed the available medical resources. Criterion C2 would lead to a high risk, with a second outbreak that would be more severe than the first one. Criterion C3 would lead to a low re-opening risk, while Criterion C4 could guarantee safe re-opening. When q equalled 30%, re-opening using relaxed criteria C1 and C2 would not be acceptable. Re-opening using criterion C3 would lead to a low risk, but re-opening with criterion C4 would be quite safe. In the case of q being 20%, re-opening with criteria C1, C2, or C3 would not be acceptable. Even with criterion C4, the strictest epidemiology criterion, re-opening would be high risk.

**Table 5:** Risk of re-opening under different epidemiological criteria and various contact tracing intensities

	q = 60%	q = 50%	q = 40%	q = 30%	q = 20%
C1	No risk	Low risk	Unacceptable risk	Unacceptable risk	Unacceptable risk
C2	No risk	Low risk	High risk	Unacceptable risk	Unacceptable risk
C3	No risk	No risk	Low risk	Low risk	Unacceptable risk
C4	No risk	No risk	No risk	No risk	High risk

One major finding of our research is that contact tracing is of significant importance when establishing re-opening policies. Track-trace-quarantine is an effective approach to breaking the transmission chain and thus limiting the risk of another outbreak [8, 30]. Digital contact-tracing Apps can fasten the contact tracing process by building a memory of proximity contacts and immediately notifying contacts of positive cases and help to achieve epidemic control [30]. This study shows that if the authorities in Wuhan could have identified and quarantined more than 60% of the close contacts of infected persons, they could have allowed the city to re-open sooner. Previous studies have shown that on average, 80% of close contacts can be traced and quarantined in many regions in China [9, 28]. However, track-and-trace measures also have capacity issues. When the size of the infected population is very large, it would be difficult or even impossible to trace all close contacts. In China, the government has put a great deal of effort into building its contact tracing capacity. For example, due to the surge in contact tracing tasks after the beginning of the COVID-19 outbreak, the Shanghai CDC had to recruit and train many ad hoc teams from local hospitals and communities to work together with its contact tracing team. New technology, such as QR code registration for public places, has been implemented to improve close contact tracing efficiency. Therefore, to ensure a safe re-opening, the government should put effort into contact tracing.

After China re-opened its economy, new confirmed cases were occasionally detected in Beijing, Qingdao and other places. Due to the strong implementation of its track-trace-quarantine policies, the government has been able to control the source of infection and stop the spread of the epidemic at the earliest possible time. To date, although many nations have started vaccinating their populations, we are not sure when COVID-19 vaccines will become widely available worldwide. A study published in Science predicted that we might have to cope with COVID-19 until 2025 [38]. Therefore, we may enter a “new normal” condition, living with COVID-19 around us, and experiencing occasional, scattered outbreaks. Under such conditions, tracing close contacts becomes even more important. It is a more efficient and cost-effective response than allowing more infections or adopting the large-scale testing of hundreds of thousands of people.

Second, our research shows that deciding the timing of re-opening using only epidemiological criteria is very risky. Without a proper level of contact tracing, re-opening will cause a severe second outbreak even when the number of new confirmed cases is very low before re-opening. In the case of Wuhan, even if the number of new confirmed cases had reached zero after 14 days with continuous decreases before re-opening, a further outbreak would still have occurred after a period of time if only 20% of the close contacts could be identified and quarantined. The new confirmed cases would increase exponentially over time due to the reinforcing loop in the SEIHR(Q) model.

Finally, hospital capacity is an additional factor that needs to be considered when deciding an administrative district’s readiness to re-open [39, 40]. Given a predicted peak value of hospitalized cases in a potential second outbreak after re-opening, the bottom line is that this value should not exceed the capacity of the health system in an administrative district. The simulation results show that if the intensity of contract tracing had been less than 30% in Wuhan, a severe second outbreak would have occurred that would have exceeded the capacity of existing hospital facilities. It is obvious that Wuhan was not ready to re-open under limited track-and-trace conditions.

The risk of re-opening summarized in Table 5 is based on the case of Wuhan, in which there was no SARS-CoV-2 variants involved, resulting in a constant transmission probability and incubation time in the model. However, to date, several new variants of SARS-CoV-2 have been found, and the WHO recently revealed the names of these variants first identified in the UK, India, and other places [41]. With new variants, the transmission probability and the incubation period, which are related to model parameters β and σ, respectively, could both change. Under such circumstances, the safe re-opening policies derived from the current model might no longer be safe because the new variants of SARS-CoV-2 could increase the transmissibility. To further investigate re-opening policies with these new variants, the related parameters should be adapted to determine which track-and-trace measures and epidemiological criteria would lead to a safe re-opening.

This model could be applicable in other countries or other settings, as long as the parameter values could be set according to the local conditions. Many differences exist among various countries, such as the geographic setting and travel patterns. Most Western countries have different political realities than Wuhan, in that compliance with track-trace-quarantine policies may be lower, even if such compliance is mandatory. The unique geographic, social and political realities in each jurisdiction would not change the basic structure of our model. However, the parameter settings and initial values would need to be adjusted to reflect these features to yield locally applicable policies for re-opening.

## Conclusions

The epidemiologic criteria, the effectiveness of track-and-trace measures, and the availability of medical resources are important factors to consider when determining the risk of further outbreaks of an epidemic after re-opening. To ensure early and safe re-opening, decision-makers need to consider all these factors when generating the re-opening policies. This study conducted a case study of COVID-19 in Wuhan and quantitively evaluated the risk of further outbreaks under various re-opening policies using the SEIHR(Q) model. Our study shows that track-and-trace measures are critically related to the level of risk associated with re-opening. There would be no risk associated with re-opening with comprehensive contact tracing in place. With partial contact tracing, re-opening would lead to a minor second wave of the epidemic. However, with only limited contact tracing, a more severe second outbreak of the epidemic would occur, overwhelming the available medical resources. In addition, epidemiological criteria could affect the risk associated with re-opening, given that the ability to implement track-trace-quarantine interventions is usually pre-defined. Different levels of risk associated with re-opening arise under various epidemiological criteria with a fixed level of track-and-trace capability.

The SD model designed in this study illustrated that the comprehensive trace-track policy implemented in China is effective for determining the conditions needed for a safe re-opening. It can serve as a micro-environment to test various re-opening policies, facilitating decision-making regarding re-opening during an ongoing epidemic.

This study has several limitations. The first concerns the assumption of the independence between the percentages of close contacts who could be traced and the number of new confirmed cases. We have not considered the fact that the percentage of contacts that could be traced decreases when the number of new confirmed cases increases sharply due to the fact that there would be an insufficient workforce to identify and monitor contacts and insufficient facilities in which to quarantine contacts. Although such a simplifying assumption is common in modelling studies of this type [39, 42], the consequence is that the SEIHR(Q) model might underestimate the risk associated with re-opening in an administrative district. The second limitation is that only epidemiological criteria, the intensity of contact tracing, and the number of medical beds were addressed in the policy simulation. Given that the WHO outlined six recommendations for rolling back lockdown, future studies could apply or extend the SEIHR(Q) model to conduct quantitative analyses that include other IPC measures and provide a more comprehensive assessment of preparedness to re-open. Finally, given the various reported values of generation interval, serial interval and incubation period, there are suggestions that real-time estimations of these parameters allowing for variations over time, should be conducted to provide more accurate estimates of reproduction numbers [34]. Tang et al. argue that using longer generation interval leads to an overestimation of the reproductive number and exaggerates control effectiveness in the initial epidemic phase [33]. Accordingly, further research can explore comprehensive evaluations of these parameters to improve the accuracy the estimated re-opening risks [34].

## Data Availability

Epidemiological data used in the current study are retrieved from the National Health Commission of the People’s Republic of China at http://www.nhc.gov.cn/xcs/yqfkdt/gzbd_index.shtml and the Health Commission of Hubei Province at http://wjw.hubei.gov.cn/bmdt/dtyw/ which are publicly accessible. All datasets are available from the corresponding author on reasonable request.

## Abbreviations

COVID-19: Coronavirus disease 2019
IPC: Infection prevention and control
WHO: World health organization
SEIHR(Q): Susceptible-exposed-infected-hospitalized-removed with and without quarantine
SD: System dynamics
